# Influence of frailty on health-related quality of life in pre-dialysis patients with chronic kidney disease in Korea: a cross-sectional study

**DOI:** 10.1186/s12955-015-0270-0

**Published:** 2015-05-29

**Authors:** Suk Jeong Lee, Heesook Son, Sug Kyun Shin

**Affiliations:** Red Cross College of Nursing, Chung-Ang University, 84 Heukseok-ro, Dongjak-gu, Seoul South Korea; National Health Insurance Cooperation Ilsan Hospital Clinical Professor, Yonsei University Medical College, Seoul, South Korea

**Keywords:** Frailty, Pre-dialysis patients, Quality of life

## Abstract

**Background:**

Chronic kidney disease (CKD) is a progressive and lifelong condition with multiple medical comorbidities. Patients with CKD experience frailty more frequently and have lower health-related quality of life than do those with other chronic diseases. The purpose of this study was to examine the prevalence of frailty and investigate the contribution of frailty to quality of life in pre-dialysis CKD patients in Korea.

**Methods:**

Using a cross-sectional survey design, data were collected at an outpatient CKD clinic in a general hospital in Korea. The frailty criterion was modified from previous studies. The Short Form-36 Health Survey version 2 was used to measure physical and mental component summary scores. Data were analyzed using chi-square, t-tests, and hierarchical linear regression.

**Results:**

Of the 168 CKD patients, 63 (37.5 %) were frail. Frail patients were significantly older and had lower physical and mental quality of life than those who were non-frail. In hierarchical regression evaluating the influence of frailty on physical and mental quality of life, the initial model was significantly improved when frailty was included. Frail patients had lower physical and mental quality of life.

**Conclusions:**

Frailty affected both physical and mental quality of life in pre-dialysis patients with CKD. More attention should be paid to the potential role of early detection and prevention of frailty to improve patients’ quality of life.

## Background

As a progressive and lifelong condition with multiple medical comorbidities, chronic kidney disease (CKD) is a global concern, and it affects approximately 5.9 % of the Korean population [1]. Frailty is defined as cumulative declines across multiple physiologic symptoms including muscle weakness, low gait speed, unintentional weight loss, exhaustion, and low physical activity [2–4]. The concept of frailty was developed in geriatric populations [4], but patients with chronic disease also suffer from frailty. In particular, CKD patients experience frailty more frequently than do individuals with normal renal function [5] and those with other chronic diseases such as cancer and vascular disease [6]. The prevalence of frailty in CKD patients ranges from 7 % [7] to 42.6 % [8], and various factors, including protein energy wasting, anemia, acidosis, and hormonal disturbances, are thought to be related to frailty occurrence in CKD patients [9].

Frailty is associated with adverse outcomes in CKD patients, such as increased risk of death [3, 6] and hospitalization [10], which leads significant impairment in health-related quality of life (HRQOL) [11, 12]. HRQOL of CKD patients differs by disease stage. Pre-dialysis patients with CKD have better HRQOL than do dialysis patients [13] and end-stage CKD patients [14], but worse HRQOL than normative adults [13] and those with other chronic diseases such as chronic obstructive pulmonary disease, asthma, and diabetes [14]. The separate influences of frailty and quality of life on CKD patients who are on dialysis treatment is relatively well documented, but few studies have examined both frailty and HRQOL. Further, little is known about the influence of frailty on HRQOL in pre-dialysis patients with CKD. The purpose of this study was to compare the factors that influence frailty and to investigate the contribution of frailty to the HRQOL of pre-dialysis CKD patients in Korea.

## Methods

### Study procedure

A cross-sectional survey with a convenience sampling method was used. The study was conducted at an outpatient CKD clinic in a general hospital in Korea from March to September 2014. The eligibility criteria for participation were as follows: diagnosis of stages 2–4 CKD, 20 years of age or older, and the ability to read and complete the questionnaire. To assess cognitive impairment, the physician of the CKD clinic who saw the patient on a regular basis conducted a primary screening to determine if the patient was cognitively able to participate in the study and provided a list of eligible participants to a research assistant. During the interview process, the research assistant conducted secondary assessments to ensure that the respondents were not cognitively impaired and were able to complete the questionnaires. In terms of ethical considerations, Institutional Review Board approval was received from the participating hospital prior to data collection. The research assistant informed participants of the purpose of the study and confidentiality. Participants were also informed of their right to withdraw from the study at any time, and written consent forms were collected from participants. The research assistant helped participants complete the questionnaires, and the questionnaire completion time was approximately 20–25 min. Using G*power version 3.0, a total of 166 individuals were estimated for multiple regression analysis with nine predictors at the alpha level of .05 and medium effect size of .15. Questionnaires were completed by 170 participants for a period of 6 months. After two individuals who failed to complete all questions were excluded, a total of 168 respondents were included in the final analysis.

### Measures

#### Frailty

The criterion of frailty was derived from three studies [8, 10, 15] and was modified for the current study. A score below 75 on the physical functioning scale of the Short Form-36 Health Survey (SF-36) was used as a marker of muscle weakness, while a score below 55 on the vitality scale of the SF-36 was used as a cut-off point for exhaustion. Patients who reported that they “almost never or never” exercised were classified as being inactive. As opposed to previous studies [8, 10] that categorized undernourishment as unintentional weight loss over 5 kg, we considered undernourishment as unintentional weight loss over 3 kg, which was previously used in Chinese elderly individuals [15]. With regard to weight loss, we assumed that the criterion of weight loss that was applied to the same ethnicity (i.e., Asian) would be more appropriate and valid, as Asians generally have a smaller build than Westerners [16]. Those who scored less than 75 on the physical functioning scale of the SF-36 were given 2 points, whereas those who scored less than 55 on the vitality scale, were in the category of exercising almost never or never, and those with unintentional weight loss over 3 kg were given 1 point each, for a total of 5 possible points. Consistent with the criterion used in Mansur et al.’s study [8], patients scoring over 3 were classified as being frail.

### Health-related quality of life

HRQOL was assessed using the SF-36 version 2 questionnaire [17], which consists of 36 items with 8 subscales. For the current study, two sets of scores, the physical component summary (PCS) and mental component summary (MCS),were used to measure HRQOL. The PCS score was calculated based on physical functioning, role limitations due to physical problems, body pain, and general health perception, while the MCS score was calculated based on role limitations due to emotional problems, social functioning, mental health, and vitality. Higher scores on the PCS and MCS indicate better HRQOL.

### Demographic and clinical characteristics

Patients’ demographic characteristics were obtained using a self-report questionnaire. Clinical characteristics including cause of CKD; comorbidities of diabetes, hypertension, or cardiovascular disease; blood pressure; blood urea nitrogen (BUN); creatinine; and hemoglobin level were collected from medical records. The level of estimated glomerular filtration rate (GFR) was calculated using the original Modification of Diet in Renal Disease (MDRD) formula [18]. Estimated GFR (eGFR) of 60–89 mL/min/1.73 m^2^ indicated stage 2 CKD, eGFR of 30–59 mL/min/1.73 m^2^ indicated stage 3 CKD, and eGFR of 15–29 mL/min/1.73 m^2^ indicated stage 4 CKD.

Based on the literature review, the variables of demographic and clinical characteristics related to frailty or HRQOL were selected for regression analyses: age [5, 8, 19, 20], gender [8, 10, 21], comorbidities [3, 19], renal function [3, 6, 7, 22], educational level and living arrangement [19, 23], employment status [24], and living with spouse [25].

### Statistical analysis

Data were analyzed using PASW Statistics version 20 (SPSS Inc., Chicago, IL, USA). The chi-square test was used to test the relationship between the categorical variables and the frailty versus non-frailty groups. For continuous variables including age, BUN, creatinine, blood pressure, hemoglobin level, PCS, and MCS, independent t-tests were conducted to compare the mean differences between the frail and non-frail groups. Hierarchical linear regression was performed to identify the influence of frailty on PCS and MCS. The initial model included gender, age, education level, living with spouse, comorbidity with hypertension, comorbidity with diabetes, employment status, and eGFR. Then, frailty was added to the second model to examine the contribution of frailty to the covariates of the previous model on PCS and MCS.

## Results

### Demographics, clinical characteristics, and physical and mental component scores between frailty and non-frailty groups

Table 1 shows the demographic, clinical characteristics, and PCS/MCS scores between frail and non-frail patients. Of 168 respondents, 63 patients (37.5 %) were frail. Frail patients were significantly older than those who were non-frail [t (166) = 2.67, *p* = .01]. Patients with hypertension etiology of CKD experienced frailty more frequently than did those with other etiologies (*χ*^2^ (1) = 3.85, *p* = .05). The mean scores of PCS [t (88.0) = 8.77, *p* < .001] and MCS [t (84.18) = 5.44, *p* < .001] were significantly lower in frail patients than they were in non-frail patients, indicating that frail patients had lower physical and mental quality of life. However, gender, educational level, marital status, CKD stage, average income, blood pressure, BUN, creatinine, and hemoglobin level were not associated with frailty.

**Table 1:** Demographics, clinical characteristics, and physical/mental component scores (*n* = 168)

	Frail (*n* = 63)		Non-frail (*n* = 105)		p
	Mean (SD)	Freq (%)	Mean (SD)	Freq (%)	
Age	69.5 (13.9)		63.7 (13.5)		.01
Gender					
Male		35 (55.6)		71 (67.6)	.12
Female		28 (44.4)		34 (32.4)	
Education					.28
≤ High school		43 (68.3)		63 (60.0)	
> College		20 (31.7)		42 (40.0)	
Marital status					.33
Married		45 (71.4)		82 (78.1)	
Other		18 (28.6)		23 (21.9)	
CKD stage (mL/min/1.73 m^2^)	38.7 (14.1)		42.6 (16.8)		.26
II (60–89)		6 (9.5)		18 (17.1)	
III (30–59)		42 (66.7)		58 (55.2)	
IV (15–29)		15 (23.8)		29 (27.6)	
Employment status					.10
Unemployed		44 (71.0)		61 (58.1)	
Employed		18 (29.0)		44 (41.9)	
Average income (10,000 won)					.06
≤100		44 (72.1)		59 (57.3)	
>101		17 (27.9)		44 (42.7)	
Etiology of CKD					
Diabetes		17 (27.0)		37 (35.2)	.27
Hypertension		26 (41.3)		28 (26.7)	.05
Glomerulonephritis		3 (4.8)		14 (13.3)	.08
Unknown		24 (38.1)		32 (30.5)	.31
Comorbidities					
Diabetes		26 (41.3)		46 (43.8)	.75
Hypertension		53 (84.1)		75 (71.4)	.06
Cardiovascular disease		8 (12.7)		10 (9.5)	.52
Blood pressure					
Systolic	127.7 (16.8)		125.9 (13.3)		.49
Diastolic	71.8 (13.0)		72.9 (10.6)		.55
BUN	25.5 (9.5)		27.4 (10.8)		.25
Creatinine	1.8 (.7)		1.8 (.7)		.96
Hemoglobin	12.2 (1.9)		12.2 (2.0)		.90
PCS	38.9 (9.6)		50.6 (5.5)		< .001
MCS	44.1 (13.6)		54.2 (7.2)		< .001

*CKD* Chronic Kidney Disease; *PCS* Physical Component Summary; *MCS* Mental Component Summary; *BUN* Blood Urea Nitrogen

### Influence of frailty on health-related quality of life

Table 2 presents the result of the hierarchical model evaluating the influence of frailty on PCS and MCS. Model 1 predicted PCS [F (8, 155) = 2.98, *p* = .004], explaining 13.3 % of the variance in PCS scores. Older patients had lower PCS than did younger patients after controlling for covariates (*p* = .002). Model 2 was significantly improved when frailty was included (R^2^ change = 29 %, *p* < .001), explaining 42.3 % of the variance. Frailty was the only significant predictor of PCS, indicating that frail patients had lower PCS (*p* < .001). Table 3 reports the results of the hierarchical model for MCS. The initial model did not predict MCS (*p* = .306). Once frailty was included, however, the second model was significantly improved (R^2^ change = 21.3 %, *p* < .001), explaining 27.1 % of the variance in MCS. Age and frailty were significant predictors of MCS, indicating that younger patients (*p* = .005) and frail patients (*p* < .001) had lower MCS after controlling for covariates.

**Table 2:** Hierarchical regression of the influence of frailty on PCS

	Predictors	β	t	p
Model 1 (R^2^ = .133, *p* = .004)	Female	-.155	−1.749	.082
	Age	-.265	−3.101	.002
	College graduate	.027	.330	.742
	Living with spouse	.108	1.328	.186
	Diabetes (Yes)	-.060	-.762	.447
	Hypertension (Yes)	-.088	−1.124	.263
	Employed	-.020	-.239	.812
	eGFR (mL/min/1.73 m^2^)	.063	.759	.449
Model 2(R^2^ = .423, R^2^ change = .29, *p* < .001)	Female	-.063	-.859	.392
	Age	-.124	−1.732	.085
	College graduate	.021	.312	.756
	Living with spouse	.081	1.219	.225
	Diabetes (Yes)	-.079	−1.225	.222
	Hypertension (Yes)	-.030	-.469	.640
	Employed	.051	.757	.450
	eGFR (mL/min/1.73 m^2^)	.040	.590	.556
	Frailty (Yes)	-.566	−8.792	< .001

*PCS* Physical Component Summary; *eGFR* Estimated Glomerular Filtration Rate

**Table 3:** Hierarchical regression of the influence of frailty on MCS

	Predictors	β	t	p
Model 1(R^2^ = .058, *p* = .306)	Female	-.026	-.285	.776
	Age	.108	1.213	.227
	College graduate	.015	-.176	.861
	Living with spouse	.165	1.980	.053
	Diabetes (Yes)	.011	.134	.894
	Hypertension (Yes)	.022	.264	.792
	Employed	-.043	-.507	.613
	eGFR (mL/min/1.73 m^2^)	.115	1.340	.182
Model 2(R^2^ = .271, R^2^ change = .213, *p* < .001)	Female	.053	.638	.524
	Age	.228	2.836	.005
	College graduate	-.020	-.269	.789
	Living with spouse	.142	1.901	.059
	Diabetes (Yes)	-.005	-.074	.941
	Hypertension (Yes)	.071	.983	.327
	Employed	.017	.228	.820
	eGFR (mL/min/1.73 m^2^)	.096	1.261	.209
	Frailty (Yes)	-.485	−6.709	< .001

*MCS* Mental Component Summary; *eGFR* Estimated Glomerular Filtration Rate

## Discussion

As compared to normative adults or patients with other chronic diseases, CKD patients experience frailty more frequently and have decreased quality of life. Little is known about the influence of frailty on HRQOL for pre-dialysis patients. Accordingly, the current study examined the prevalence of frailty and the contribution of frailty to HRQOL in pre-dialysis CKD patients in Korea. Our study found that older age was associated with a higher prevalence of frailty, which is consistent with other studies [5, 8, 19]. We also found that the patients with hypertensive etiology of CKD experienced frailty more frequently. The mechanism underlying the relationship between hypertension etiology and frailty is unclear, but subclinical vascular disease, such as hypertension, may contribute to the greater likelihood of frailty in patients with CKD [6]. Further, regardless of the etiology of CKD, there was no difference in diastolic and systolic blood pressure between frail and non-frail groups, which makes the influence of hypertension on frailty inconclusive. Further studies are necessary to determine the association between hypertension etiology and frailty in pre-dialysis CKD patients.

Similar to another study [19], educational level and living alone were not associated with frailty in the current study. In contrast, there are inconsistent findings with regard to the relation between gender and frailty. We found that frailty was not different between men and women, but female patients were found to be more frail in other studies [8, 10]. This discordance may be due to different sample characteristics. In Johansen’s study [10], the study population was composed of hemodialysis patients, which may suggest that the influence of gender on frailty depends on renal function. Although Mansur’s [8] study and the current study included pre-dialysis patients, the relation between gender and frailty might be explained by a third variable. In addition to gender, for example, age might play a role in the relation with frailty. In the current study, women were younger than men were. The mean age in women and men was 62.5 and 67.8 years, respectively, and the proportions of older age (≥60 years) in women and men were 54.8 % and 72.7 %, respectively (data not shown). Frailty increases with age, such that aging is related to progressive homeostatic dysregulation, which then leads to non-resilient conditions [26]. It may be true that women are more likely to be frail if the potential confounding factor of age is taken into account. In our study, however, the influence of gender on frailty might have weakened because age was not comparable between men and women. Therefore, future studies are necessary to support the relationship between gender, age, and frailty.

It has been consistently reported that CKD patients who are older [5, 8, 19] and who have lower renal function [3, 6, 7] are more vulnerable to frailty. Regarding the influence of age and renal function on frailty, the severity of CKD might have a greater impact on frailty than age does. Renal function, age, and the prevalence of frailty differed in the current study and Johansen’s study [10]. The prevalence of frailty was 67.7 % and 37.5 % in Johansen’s study and the present study, respectively. Our study included pre-dialysis patients with stages 2–4 CKD, while Johansen’s study [10] targeted stage 5 dialysis patients, which means that the renal function of the patients in our study was better than it was in Johansen’s study. On the other hand, the frail patients in our study were older than were those in Johansen’s study (65.9 vs. 58.2 years old, respectively), but the prevalence of frailty was lower in the current study. Consequently, age influences frailty, but renal function also greatly influences frailty in CKD patients.

It is known that frailty is closely related to quality of life, but few studies have examined the relationship between frailty and quality of life simultaneously. Most studies have reported the two concepts of frailty and quality of life separately. We found that physical and mental quality of life were significantly lower in frail patients as compared to non-frail patients, which is congruent with studies examining frailty and quality of life in older adults [15] and in pre-dialysis CKD patients [8]. In hierarchical regression for the outcome variable PCS, frailty significantly contributed to improvement of the model. After adjusting for the covariates, the variance increased when frailty was included, indicating that frailty greatly influences physical health in pre-dialysis CKD patients. Age might also influence physical HRQOL. In the initial model without frailty, old age predicted worse PCS, which is consistent with other studies [21, 22]. After frailty was included in the first model, however, age was no longer significant, and only frailty predicted PCS, indicating that frail patients had lower PCS. This finding may support the claim that the influence of frailty on PCS is critical, and that frailty is an independent, significant predictor over age.

Frailty was also related to decreased mental quality of life. When frailty was included into the initial model, the variance of MCS increased and age became significant, indicating that younger patients had lower MCS. On the other hand, other studies [21, 23] showed inconsistent results indicating a relationship between older age and lower MCS. The concept of frailty was only included in the current study, whereas frailty was not measured in the other two studies. Thus, it appears that frailty contributes to this discrepancy, indicating that there may be an interaction between frailty and age on MCS. In the subgroup analysis (data not shown), younger patients (age < 60 years) had lower MCS than older patients (age ≥ 60 years), regardless of frailty. Regarding age group and frailty, the MCS scores were as follows: 42.58 in the young frail group, 44.67 in the old frail group, 50.41 in the young non-frail group, and 56.28 in the old non-frail group. Thus, MCS scores were lowest in the young frail group. The score of 42.58 in the young frail group was lower than that found in stage 4 CKD patients [22]. Further, participants’ age and renal function might contribute to this discrepancy. The mean age of participants in our study was 65.9 years, while the patients in other studies were relatively younger, namely, 51.7 years [23] and 54.4 years [21]. The study participants in the two studies also had more advanced renal condition in terms of pre-dialysis vs. dialysis patients, indicating that disease severity may be associated with mental quality of life.

There are several weaknesses of the current study. First, the cross-sectional study design limits the ability to determine causal relationships. Additionally, the findings may not be generalizable to pre-dialysis CKD patients not included in this study. In addition, cognitive impairment of the patients was assessed by the physician and research assistant. Although the assessment was done by the professional and the interviewer in two steps, it may still be possible that the screening was biased as opposed to using a valid assessment instrument measuring cognitive impairment. Despite these limitations, our study highlights the significant contribution of frailty to HRQOL in CKD patients. Although pre-dialysis patients have a better quality of life compared to those on dialysis, they have still have a lower quality of life than those without CKD or patients with other chronic diseases [27].

We found that frail patients had lower HRQOL than did non-frail patients. In particular, as MCS decreases, repeated psychological distress occurs, which leads to impaired homeostatic equilibrium and emergence of disease [28]. Given that CKD is a lifelong disease, it is important for pre-dialysis CKD patients to provide interventions that increase quality of life. For example, a nutrition intervention improved quality of life in pre-dialysis CKD patients [29]. Various factors, including inflammatory factors, hormone changes, malnutrition, anemia, and depression, have been known to influence the occurrence of frailty [30, 31]. As a high proportion of CKD patients experience frailty that influences long-term quality of life, early frailty detection and preventive intervention programs are necessary to improve quality of life. Exercise training for pre-dialysis CKD patients improved physical impairment and health-related quality of life [32], which could be a good example.

## Conclusions

The present study showed that frailty influenced both physical and mental quality of life in pre-dialysis patients with CKD in Korea. More attention should be paid to the potential role of early detection and prevention of frailty to improve patients’ quality of life. Further studies are necessary to develop effective interventions to address modifiable factors influencing frailty and quality of life among patients with CKD.

## Abbreviations

CKD: Chronic kidney disease
HRQOL: Health related quality of life
SF-36: Short form-36 health survey
PCS: Physical component summary
MCS: Mental component summary
GFR: Glomerular filtration rate
BUN: Blood urea nitrogen
